# Assessing the impact of the four COVID-19 variants and the vaccine coverage on mortality in Malta over 2 years: An observational case study

**DOI:** 10.3389/fpubh.2022.1018505

**Published:** 2022-09-23

**Authors:** Sarah Cuschieri, Stephan Grech, Victor Grech

**Affiliations:** ^1^Department of Anatomy, University of Malta, Msida, Malta; ^2^Mater Dei Hospital, Msida, Malta

**Keywords:** COVID-19, mortality, vaccination, disease burden, mutation, Malta

## Abstract

**Background:**

Mortality may quantify a population's disease burden. Malta, like other European countries, experienced COVID-19 surges in cases and mortality across the pandemic. This study assesses COVID-19's mortality impact, while exploring the effects of the four dominant COVID-19 variants and that of the vaccination coverage on the Maltese population.

**Methods:**

COVID-19 data (cases, mortality, positivity, and vaccination rates) was obtained from the websites of the European Center for Disease Prevention and Control and the Malta Ministry of Health. Data was categorized into the four periods according to reported dominant COVID-19 variant. Years of life lost (YLL) and Case-Fatality-Ratio (CFR) for each period were estimated. CFR was also estimated for the pre-vaccine and post-vaccine periods.

**Results:**

The original COVID-19 period (36 weeks) had the highest YLL (4,484), followed by the Omicron variant period (12 weeks; 1,398). The Alpha variant period (7 weeks) had the highest CFR (1.89%) followed by the Original COVID-19 (1.35%). The pre-vaccine (1.59%) period had higher CFR than the post-vaccine period (0.67%).

**Conclusion:**

Various factors contributed to mortality, but the variant's infectivity, transmissibility, and the effectiveness of the vaccine against the variant play an important role. Reducing mortality by embracing mass vaccination that targets current variants along with other non-pharmaceutical interventions remains paramount.

## Introduction

The novel coronavirus SARS-CoV2 was first reported in Wuhan, China at the end of 2019 and within weeks spread globally, resulting in the COVID-19 pandemic (1). During the pandemic various mutations occurred to the original viral strain resulting in the emergence of several variants of interest and variants of concern (2). The small Mediterranean islands of Malta with a total population of 514,564, like the rest of Europe, were affected by COVID-19. The first COVID-19 case reported in Malta was in March 2020 and for a whole year the original SARS-CoV2 virus dominated the island's population (3). The Beta variant (B.1.351) was first detected in Malta in February 2021 although only a couple of cases were identified before the Alpha variant (B 1.1.7) took over the scene from March 2021 (4, 5). This led to a spike in cases bringing about Malta's second lockdown (6). At the time, COVID-19 vaccination rollout was well underway with a substantial proportion of the Maltese elderly population fully vaccinated, while the younger age groups were progressively being inoculated (7). June 2021 saw the first case of the Delta variant (B.1.617.2) in Malta, which became the dominant variant across the islands within weeks (8). The new surge in cases and mortality led to the initiation of the booster dose vaccination rollout targeting the elderly in September 2021 (9). The first Omicron variant (B.1.1.529) cases were reported during the end of December 2021 and in days became the new dominant variant (10, 11). By the end of February 2022, total reported COVID-19 cases since the onset of the pandemic in Malta were 13,308 cases per 100,000 population and 126 per 100,000 population deaths (12).

Mortality is an important index for quantifying the burden of a disease among the population and also constitutes a fundamental pillar for public health decision making (13, 14). In this study, we set to assess the impact of COVID-19 in terms of population mortality, while exploring the effect of the four dominant variant phases and the vaccination coverage on the Maltese population. The small population size of Malta provides a unique opportunity to evaluate the burden of COVID-19 at a population level, and the evidence generated by this exercise is of importance to both local and international public health authorities and policymakers in their role in the prevention and control of the ongoing pandemic.

## Methods

This observational study was based on freely available epidemiological data and public health announcements reported in local newspapers from the onset of COVID-19 till the end of February 2022. The European Center for Disease Prevention and Control (ECDC) database (https://www.ecdc.europa.eu/en/covid-19/data) was utilized to obtain Malta's COVID-19 data for weekly cases, positivity rate and vaccination rate stratified by age and gender. The Ministry of Health official repository (https://github.com/COVID19-Malta/) was used to obtain the daily mortality data stratified by age and gender. Excess mortality data was obtained from the Eurostat website (15).

Weekly cases, positivity, mortality, and vaccination data were categorized according to these four phases: (i) Original COVID-19^*^ from week 30 of 2020 to week 14 of 2021; (ii) Alpha variant from week 15 of 2021 to week 22 of 2021; (iii) Delta variant from week 23 of 2021 to week 48 of 2021; (iv) Omicron variant from week 49 of 2021 to date (end of February 2022). For this study's analyses, Original COVID-19 phase^*^ was considered to start from week 30, i.e., with the onset of the second wave since during the first wave, the COVID situation in Malta was well-controlled with low positive cases and deaths (3). It needs to be noted that only the dominating variant in a particular phase was considered for the purpose of the study analyses, but this does not preclude that a small proportion of cases and deaths were due to different variant/s.

### Data analyses

#### Year of life lost

The Years of Life Lost (YLL) is a metric used in population health to measure the number of years lost due to premature death from a particular cause. The YLL calculation provides a good comparative insight into the impact of death on the population as it recognizes deaths occurring at a younger age group as having a greater impact on population health as opposed to deaths occurring at an advanced age group (16). Following the Global Burden of Disease (GBD) Study methodology, the years of life lost (YLL) was estimated by combining the death counts by five-year age-groups and sex (17). The estimates were calculated by multiplying the number of deaths in each age-group by the age-conditional remaining life expectancy from the GBD Study 2019 reference life table, where the same values are assigned to both males and females (18). The YLL for each of the four COVID-19 variant phases was estimated using the described calculation. In view that the YLL metric considers the mortality impact over a period of a year, but the different variants were dominant for weeks, the YLL established was divided by 52 (number of weeks in a year) and then multiplied by the total number of weeks each variant dominated (Original 36 weeks; Alpha 7 weeks; Delta 26 weeks; Omicron 12 weeks). The YLL per week was also calculated i.e., YLL/52. This calculation is expected to provide an indication of the impact of a variant on premature mortality over the duration of its dominance.

#### Case-Fatality-Ratio

The Case-Fatality-Ratio (CFR in %) for ongoing epidemic was calculated using the formula below (19). The CFR for each of the four COVID-19 variant phases was estimated. The CFR was re-calculated to consider the impact of COVID-19 vaccine roll-out on mortality. Therefore, the pre-vaccine CFR (week 30/2020 till week 52/2020) and the post-vaccine CFR (week 1/2021 till week 9/2022) were also calculated. The post-vaccine period covered from the start of the first dose up till the booster dose among the study population.


$$\begin{array}{l} \text{Case fatality ratio }(\%)=\\ \frac{\text{Number of COVID}-19\text{ deaths}}{\text{Number of COVID}-19\text{ deaths + Number of recovered}}\\ \times \;100 \end{array}$$

#### Vaccination

Data on cumulative full dose vaccination, cumulative booster dose and mortality were stratified by age groups (25–49; 50–59; 60–69; 70–79; 80+ years). For trend analysis the Jonckheere-Terpstra test was used for both the cumulative full dose vaccination and cumulative booster dose against the mortality by age groups at 50% vaccination uptake at a population level. Full vaccination was estimated to have reached 50% uptake at week 21/2021 for the 25–49 years, week 23/2021 for 50–59 years; week 20/2021 for 60–69 years; week 14/2021 for 70–79 years and week 8/2021 for 80+ years. While booster dose was estimated to have reached 50% uptake at week 52/2021 for 25–49 years; week 50/2021 for 50–59 years; week 47/2021 for 60–69 years; week 42/2021 for 70–79 years and week 40/2021 for 80+ years.

## Results

**Figure 1:**
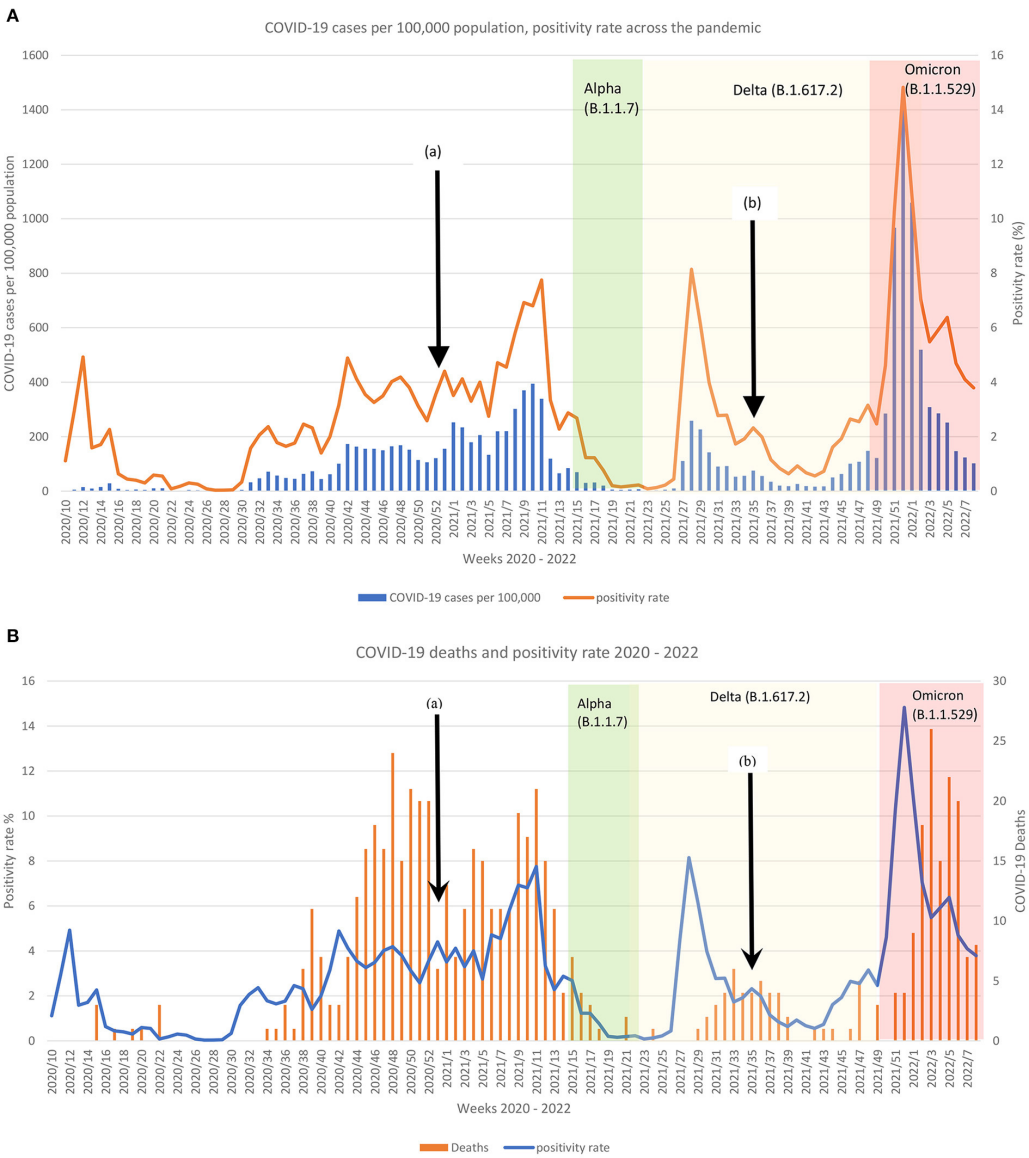
Comparison of the reported **(A)** COVID-19 cases per 100,000 and positivity rate per week and **(B)** COVID-19 deaths and positivity rate per week according to the dominating variant across 2 years in Malta. Black arrow (a) indicates start of COVID-19 vaccination and (b) start of booster dose. White background = original SARS-CoV2; Green background = Alpha variant; Yellow background = Delta variant; Red background = Omicron variant.

Across the 2 years of the pandemic, Malta reported three dominating COVID-19 variants apart from the original SARS-CoV2, leading to several surges in infection and mortality cases as shown in Figures 1A,B. Over 12 weeks, the Omicron variant appeared to have had the worse infectivity spread with an average positivity rate of 6.7 when compared to the rest of the variants (Original: dominated 36 week, average 6.7 positivity rate; Delta variant: dominated 26 weeks, average 2.10 positivity rate; Alpha variant: dominated 7 weeks average 0.84 positivity rate).

In the initial pandemic phase, the original variant had low mortality (Figure 1B), but the second wave led to a surge of deaths. Weekly deaths continued to be reported until mid-May 2021, when the Alpha variant surge subsided. During the original and delta COVID-19 phases, the surges in deaths could be observed on average 5 weeks following the spike in positive cases. The mortality rate during the Delta variant phase was much lower than that of the original COVID-19. Conversely, this was not the case following the Alpha variant surge, where mortality declined (Figure 1B). The aftermath of the Omicron variant peak led to the highest death rate over a period of a week from the onset of the pandemic (Figure 1B).

When comparing the four COVID-19 variant phases, the original COVID-19 phase contributed to the highest adjusted YLL (4,484 years), followed by the Omicron phase (1,398 years–Table 1). YLL contributed by the original COVID-19 was for a period of 36 weeks as opposed to the 12 weeks period of Omicron. A similar YLL distribution could be observed when comparing the YLL per week across the different variants, as shown in Table 1. On comparing the case-fatality ratio (CFR) of the four COVID-19 phases, the Alpha variant was observed to contribute to the highest ratio, followed by the original COVID-19 (Table 1). The CFR for the pre-vaccine period (1.59% over 22 weeks) was higher than that of the post-vaccine period (0.67% over 61 weeks). Excess mortality per month was reported throughout the pandemic and across all the four phases as shown in Supplementary Table 1.

**Table 1:** Comparison of the four COVID-19 phases epidemiological outcomes.

	**Original (36 weeks)**	**Alpha (7 weeks)**	**Delta (26 weeks)**	**Omicron (12 weeks)**
Total cases (*N*)	29,166	901	9,280	29,055
Total mortality (*N*)	393	17	49	181
Case-Fatality Ratio (CFR–%)	1.35	1.89	0.53	0.62
Years of life lost (YLL—years)	4,484	46	444	1,398
YLL/week	125	7	17	52

### Impact of vaccination coverage on mortality

**Figure 2:**
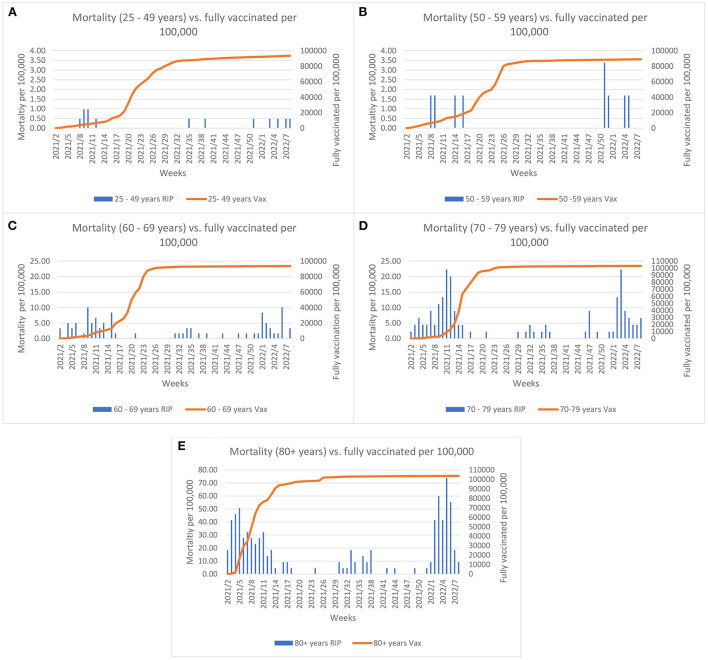
Comparisons between morality rate and full vaccination (2 doses or 1 Janssen dose) coverage per 100,000 across age groups and weeks since initiation of vaccine rollout. Left y-axis Graph **(A,B)** scale represents mortality up till 4 per 100,000; Graph **(C,D)** scale represents mortality up till 25 per 100,000 and Graph **(E)** represents mortality up till 80 per 100,000.

**Figure 3:**
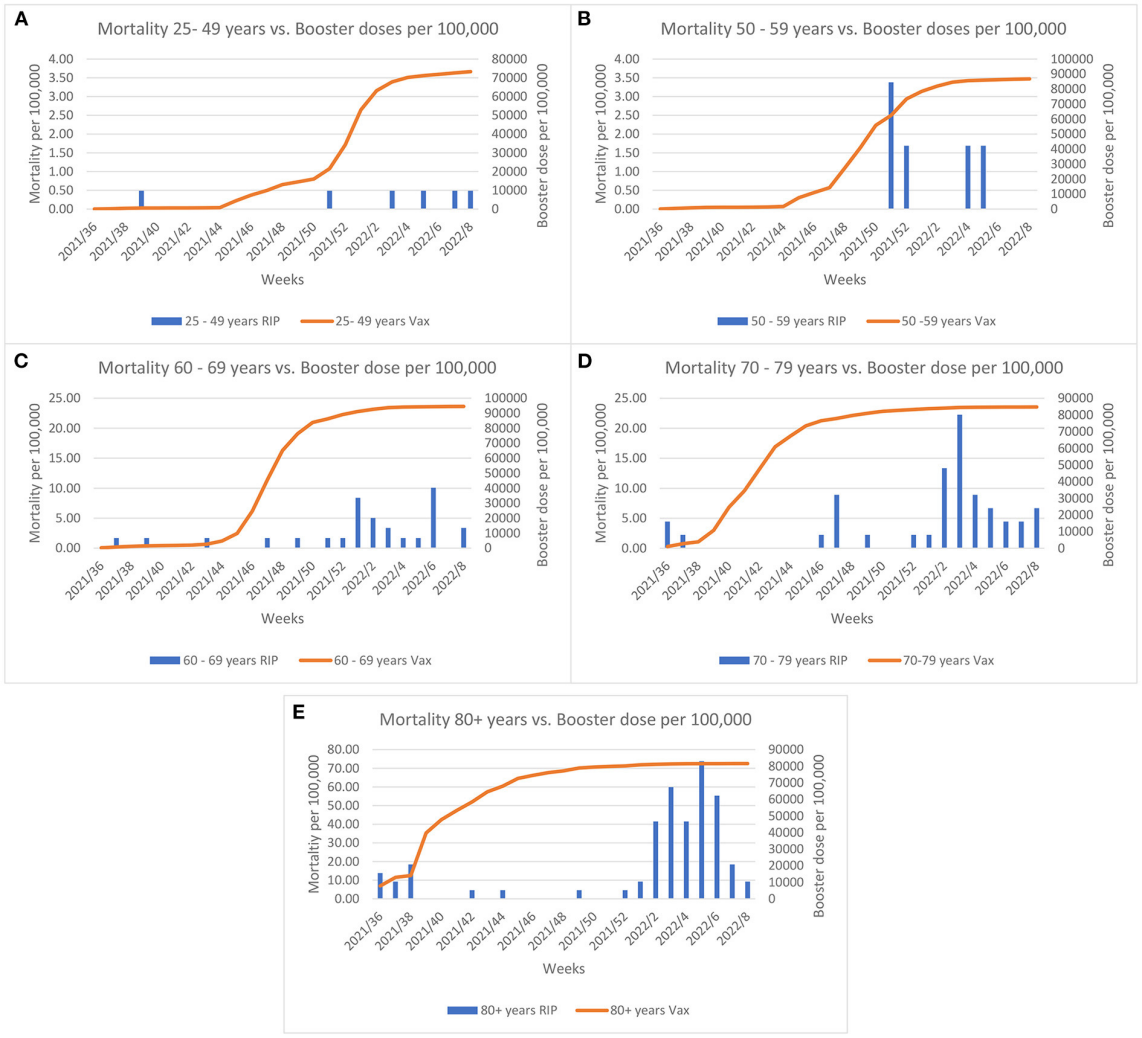
Comparisons between morality rate and the booster dose coverage per 100,000 across age groups and weeks since initiation of the booster rollout. Left y-axis Graph **(A,B)** scale represents mortality up till 4 per 100,000; Graph **(C,D)** scale represents mortality up till 25 per 100,000 and Graph **(E)** represents mortality up till 80 per 100,000.

The COVID-19 vaccine rollout started on the 27th December 2020 in Malta and by summer 2021 almost the entire eligible population had been fully vaccinated. Evaluation of the effect of vaccination coverage on the mortality rate (per 100,000 population) across the different age groups, shows that the mortality rate declined until the Omicron outbreak (Figure 2). A borderline significance was established for these trends (*p* = 0.05). The elderly were invited to take the booster dose as the Delta variant predominated at the end of summer 2021. As shown in Figure 3, when the Omicron variant was detected in Malta a large proportion of the elderly (60+ years) were already inoculated by the booster dose. Despite this, an increase in the mortality rate can be observed across all age groups, with borderline significance (*p* = 0.05).

## Discussion

Mortality is a useful measure to assess the magnitude of the pandemic as well as act as a tracking tool of the pandemic's impact on the population (20). Since the onset of the pandemic, the mortality rate has been on the incline, with certain COVID-19 phases experiencing a higher rate than others, as was observed in this study. It needs to be acknowledged that although this was out of the scope of this study, the excess mortality noted since the onset of the pandemic is not only a direct effect of the COVID-19 infection but also due to secondary indirect causes such as economic turmoil, lockdowns and pandemic related anxiety leading to higher suicide deaths among other factors (14, 21, 22).

The only preventive measures available during the first year of the pandemic were non-pharmaceutical interventions (NPI). Their success in pandemic control was dependent on timely measures instituted by the country's authorities and the population's compliance. During the first COVID-19 wave, Malta was praised for its effective pandemic management resulting in low infectivity and mortality rates (3), as supported by this study. Yet, abrupt lifting of the measures and mass gatherings brought about the second wave (23). At the time the dominant original COVID-19 variant resulted in a sharp rise in mortality, as noted in this study. Indeed, this period contributed to the highest years of life lost (YLL) out of the four variant phases, yet not the highest CFR. COVID-19 has affected individuals across all age groups with premature deaths occurring even among the young generation (24). Hence, the YLL metric provides a good indication of the COVID-19 impact on the population in terms of premature mortality. Much of the original COVID-19 disease phase relied on just NPI's for prevention, as the COVID-19 vaccine became available when the original COVID-19 began to phase out and new variants took over. This may explain the high YLL attributed to this phase as mortality occurred among young adults apart from more advanced adults. Additionally, this phase lasted for a longer duration than the other variants. Yet, despite the presence of the vaccine and the booster dose, the Omicron variant phase was observed to have the highest positivity rate and mortality occurrence at a population level as well as having the second highest YLL in this study. This may imply that mortality among the younger generation occurred even if the percentage vaccinated adults was high, although other confounding factors might also have been present. It has been reported that two doses vaccination does not provide adequate protection against the Omicron variant, while the addition of a booster dose only adds a low protective effect, with a decline in its effectiveness after some 4 months post vaccination (25, 26). This relationship could be clearly observed in this study, where despite high vaccination uptake, the mortality rate did not decline. Another feature exhibited by the Omicron variant is its ability to evade the immune system, with those having the booster still susceptible to infection (27). These Omicron features might have played a role in the rise in the mortality rate observed in this study. The elderly were inoculated with the booster between September and October 2021, with wanning immunity when the Omicron variant dominated the scene in Malta. Of note, the Omicron variant phase considered in this study was of a period of 12 weeks, as opposed to the original COVID phase (36 weeks). Therefore, with caution one might project that if the Omicron variant continuous to dominate the landscape with the same virulence level, it will lead to a higher level of premature mortality.

From the start of the pandemic, Malta, had followed a high swab testing policy and progressively increased swabbing hubs across the islands to make testing accessible to all of the population (3, 28). Therefore, calculating the case-fatality ratio can be considered as reliable measure of severity and a valuable piece of policymaking (29). When evaluating the impact of mortality in terms of CFR, the Alpha variant phase contributed to the highest CFR proportion, even though this had the shortest phase. This points to the highly transmissible feature of this variant over a short period of time along with its associated high mortality (30, 31). Despite this, a mortality decline was observed across this Alpha variant phase which corresponds to the high uptake of COVID-19 vaccination among the population. Indeed, it has been reported that the COVID-19 vaccine is highly effective in decreasing transmission and mortality vs. the Alpha variant (32). The pre-vaccine period was noted to hold a higher CFR proportion than the post-vaccine period. With caution this may indicate that vaccines had a positive effected on the overall mortality incidence even if the dominating variants during the vaccination phase resulted in a substantial high positivity and mortality rate. Although the vaccine efficacy could not be measured for this study population, other studies have reported a relationship between vaccine efficacy and a decrease in the all-cause mortality and hospitalisations irrespective of the dominating variant (33–35). This is an important public health finding as it provides evidence how the impact of future COVID-19 waves can be reduced by enhancing mass population vaccination while safeguarding the healthcare systems. However, when interpreting this study's findings, the duration of both periods need to be considered, i.e., the pre-vaccine period was shorter than the post-vaccine period, which might have had an effect to this finding, apart from other potential confounding factors. Further research is therefore recommended to investigate the effect of vaccination on mortality outcome.

Several strengths and limitations need to be acknowledged. The study was based in a small country making it easier to explore the COVID-19 impact at a population level. From the onset of the pandemic, Malta had a high swab testing capacity including testing every individual that is admitted to hospital and post-mortem (3), so the detection of COVID-19 can be considered as being representative of the population. This study was an observational study based on epidemiological data freely available through ECDC and the Maltese government repository. The authors did not have direct access to the genotyping or medical history of the infected population nor to those that died, which might have impacted on the study's outcome including the inability to perform regression analyses and other complex analyses pertaining to the different variants. Furthermore, individualized vaccination data was not available to estimate the vaccine efficacy. Other underlying confounding factors, apart from vaccination, might have influenced the mortality outcome across the four variant phases. Assumptions had to be made that once the authorities reported that a variant is dominant within the population, the recorded cases, and deaths from that point in time were affected by that same dominant COVID-19 variant. However, this may have overestimated the effect of the dominant variant as other variants might have been present. Delayed mortality reporting might have occurred possibly leading to under reporting or overreporting of deaths during a particular COVID-19 variant phase. The authorities report daily mortality but do not differentiate between individuals dying due to COVID-19 or dying while also having COVID-19. Therefore, in this study we were unable to take this in consideration.

## Conclusion

Morality data provides an indication of the burden of COVID-19 within a population. Various factors contribute to mortality, yet the variant's infectivity, transmissibility, and the effectiveness of the vaccine against the variant play an important role. The pandemic is far from over and reducing mortality should remain high up on the agenda by embracing mass vaccination that targets the current variant as well as the institution of timely preventive measures across countries.

## Data availability statement

Publicly available datasets were analyzed in this study. This data can be found at: https://www.ecdc.europa.eu/en/covid-19/data, https://www.facebook.com/sahhagovmt.

## Author contributions

SC was responsible for the design of the study, data collection, data analyses, and writing of the draft article. SG and VG were contributed to the study design and critically reviewing the article. All authors contributed to the article and approved the submitted version.

## Conflict of interest

The authors declare that the research was conducted in the absence of any commercial or financial relationships that could be construed as a potential conflict of interest.

## Publisher's note

All claims expressed in this article are solely those of the authors and do not necessarily represent those of their affiliated organizations, or those of the publisher, the editors and the reviewers. Any product that may be evaluated in this article, or claim that may be made by its manufacturer, is not guaranteed or endorsed by the publisher.
