# Supplementary material for: Assessing the impact of the four COVID-19 variants and the vaccine coverage on mortality in Malta over 2 years: An observational case study
Source: Front Public Health. 2022 Sep 23;10:1018505. doi: 10.3389/fpubh.2022.1018505 (PMC9541139; doi:10.3389/fpubh.2022.1018505)
Supplement: Supplementary file 1 [file Table_1.DOCX]

| Year-Month | Excess mortality (%) | Covid-19 mortality per 100,000 |
| --- | --- | --- |
| 2020-01 | -0.30% | 0.00 |
| 2020-02 | -7.90% | 0.00 |
| 2020-03 | 16.70% | 0.00 |
| 2020-04 | 12.60% | 0.78 |
| 2020-05 | 2.20% | 0.97 |
| 2020-06 | -4.40% | 0.00 |
| 2020-07 | 7.20% | 0.00 |
| 2020-08 | 13% | 0.58 |
| 2020-09 | 22.20% | 4.47 |
| 2020-10 | 21.10% | 5.64 |
| 2020-11 | 38.30% | 14.96 |
| 2020-12 | 40.50% | 15.16 |
| 2021-01 | 3.60% | 9.33 |
| 2021-02 | 15.70% | 0.00 |
| 2021-03 | 32.60% | 14.96 |
| 2021-04 | -0.10% | 4.08 |
| 2021-05 | 13% | 1.17 |
| 2021-06 | 1.70% | 0.19 |
| 2021-07 | 17.70% | 0.58 |
| 2021-08 | 26.80% | 3.50 |
| 2021-09 | 17.50% | 3.11 |
| 2021-10 | 12% | 0.78 |
| 2021-11 | 11.90% | 1.36 |
| 2021-12 | 16.50% | 1.75 |

Supplement Table 1. Excess mortality percentage and Covid-19 mortality per 100,000 across the four Covid-19 phases

White background = original SARS-CoV2; Green background = Alpha variant; Yellow background = Delta variant; Red background = Omicron variant
